# Obstetrics and Diseases of Women and Children

**Published:** 1861-05

**Authors:** Wm. B. Atkinson

**Affiliations:** Lecturer on Obstetrics, and Diseases of Women and Children


### OBSTETRICS, AND DISEASES OF WOMEN AND CHILDREN.

BY WM. B. ATKINSON, M.D.,
LECTURER ON OBSTETRICS, AND DISEASES OF WOMEN AND CHILDREN.

### I.—*Rigidity of the Os Uteri.* By CHARLES ARNOTT, M.D., Edinburgh.

A rigid os is, perhaps, one of the most frequent causes of lingering labor. The pains are regular, increase in severity, and reach an intensity that assures all present of a speedy delivery. Examination reveals a dry, rigid vagina; the os high up, posteriorly, with an opening barely sufficient to admit the finger; the fœtal head low down, anteriorly, covered by the uterine walls, and at each pain appearing as if about to force its way through these tissues. Thus, labor may continue for hours. In the majority of cases, the os is not truly in a rigid state, but the absence of the dilating power of the wedge-like "bag of waters," the membranes having ruptured early, produces delay, and the head is driven not directly into the os, but against the anterior wall of the uterus. Dr. Arnott regards the usual treatment of these cases as highly improper, and prefers reduction and dilatation by manipulation, or in more aggravated cases incision of the opposing texture. The parts should be freely lubricated, the finger introduced during each pain, and the os drawn gently downward and forward, and at the same time dilated. A speedy improvement is the result, though in a small minority the os may require a slight notching with the bistoury. Dr. Arnott considers this but an imitation of nature, as the practitioner frequently observes spontaneous laceration, even to a great extent, as occurring in such cases. This plan he has followed for many years, and always with benefit. Never having encountered a case where any evil resulted, he believes it almost entirely free from danger.—(*Lancet*, January, 1861.)

II.—*Placenta Prœvia; Treatment by the Caoutchouc Water Pessary.* By E. J. FOUNTAIN, M.D., Davenport, Iowa.

Mrs. P., aged twenty, at seven and a half months of her pregnancy, began to have hemorrhage, but without labor-pains or dilatation of the os. This ceased partly under the use of rest, cold water, enemata, opium, and acetate of lead. At the end of two weeks, it recurred with greater violence and some pain. Dilatation was sufficient to allow the finger to enter, and the placenta was ascertained to be in front. To check the alarming discharge, a caoutchouc bag was introduced, and filled full of cold water, which at once arrested the flow. In half an hour it again commenced moderately; the water, now quite warm, was allowed to escape, and the bag refilled. By the continuance of this process through the day and night, the patient was kept safe. When the contents were changed, an examination could be made without removing the instrument. After twenty-four hours, as the os was becoming well dilated, and the pains more regular, turning was thought of, but finally rejected, and the former plan continued. Finally the head pressed down on the placenta, thus perfectly controlling the hemorrhage, and the child was soon born in good condition, about thirty hours after the commencement of the treatment. The placenta was found loose in the vagina.—(*Am. Med. Times*, March 9, 1861.)

III.—*Quinia in Tedious Labors.* By JOHN LEWIS, M.D., Knightstown, Ind.

In the proceedings of the Union Medical Society, Dr. Lewis reports a case of labor in which, after about ten hours of slight ineffectual pains, he had the patient's feet bathed in warm water, dry cups applied to the sacrum, and gave at once ten grains of quiniæ sulphas; the pains immediately increased in frequency and duration, and the labor steadily progressed to a speedy termination. He says this has been his custom for the last ten years, and he expects the same result, as surely as nausea from ipecac. or purging from jalap.—(*Am. Med. Times*, February 16, 1861.)

IV.—*Rupture of Uterus; Recovery.* By G. T. ELLIOT, JR., M.D., New York.

On the 25th of November, 1860, I was called by Dr. Slevin to see Mrs. M. in her second labor. The first had been severe, but terminated naturally. She had suffered for eighteen hours, when she complained of a sharp, agonizing pain in the left iliac region, and the contractions ceased. Before this the brow, tips of the fingers and the funis had been recognized as presenting, but they had now receded. She was weak, and vomiting a clear green fluid; pulse one hundred and thirty, and feeble. Within the cervix, to the left, was a longitudinal fissure, which did not involve the entire thickness of the cervix. It was decided to turn; the patient took some stimulus, and the operation was completed, the delivery of the head requiring its being broken up, as locking occurred. After the delivery, the fingers could be passed through the rent so as to feel a loop of intestine and the peritoneal coat of the abdominal wall. Slight hemorrhage took place, and contraction ensued, which was aided by ergot and ice in the vagina. The ergot was vomited almost immediately. The patient was placed in bed, and care taken to procure reaction. The vomiting was persistent for two days, when she began to improve, and by the latter part of December was out walking. The treatment was solely sedative and stimulant.—(*Am. Med. Times*, February 23, 1861.)

V.—*Successful Case of Cæsarean Section.* By JAS. EDMUNDS, M.R.C.S., etc., London.

Mrs. P., aged thirty-eight, in labor for five days, became exhausted. On examination, there was found projecting into the vagina a hard mass, two inches in diameter, superficially ulcerated and tuberculated, so that the os could with difficulty be made out. When the finger was passed in, there was felt a dense, unyielding tissue, referable only to cancerous enlargement of the os and cervix. Has had for two years severe pain in coitu, and a fetid sanguinolent discharge, with constant aching and lancinating pains in the loins, hips, and thighs. Her pains are frequent, with a muco-sanguineous discharge. She was kept on good diet, opium, and patience enjoined. After the lapse of the above period, she being a calm, courageous, intelligent woman, was told of the alternatives; exhaustion and death from obstructed labor; laceration of the os uteri, and death from hemorrhage; or, on the other hand, an operation, either slitting up the os, and delivery by the forceps, or craniotomy, or the Cæsarean operation. On the sixth day of the labor, the latter was performed, after the usual preparation. The urine was drawn off, the liquor amnii discharged, and she was then seated on the edge of the bed, her legs hanging over, the head and shoulders supported by pillows, and a warm flannel petticoat loosely tied round her waist. Chloroform was administered, and with a large scalpel an incision was made upon the inner edge of the rectus muscle, from the navel down to within a short distance of the pubis. The uterus was opened from above downward, the placenta and child were quickly extracted, and the uterine fibres contracted and firmly closed the opening. The wound in the abdomen was then carefully closed by means of the interrupted suture and strips of adhesive plaster, after which the usual dressings were applied. She was placed on beef-tea, anodynes, etc. After the usual favorable and unfavorable changes, by the fourteenth day the wound was soundly consolidated, and all going on as in ordinary labor.—(*Med. Times and Gazette*, January 5, 1861.)

VI.—*The Functions of the Placenta.* By V. NIVET, D.M.P., of Clermont Ferrand.

M. Nivet, in a letter to Prof. Courty, takes issue with him on the ground that he has declared the placenta to be exclusively an organ of absorption and nutrition. M. Nivet maintains that it is also an organ of sanguification. He explains this as meaning that function which has for its purpose the adding of oxygen to the blood which traverses the capillaries of certain organs; the result is that the globules become redder and the blood warmer. This function is direct or indirect. In the direct, the black blood, which has furnished materials to the organs of secretion and nutrition, comes in contact with the atmosphere, or with a liquid containing oxygen, which gas unites with it.

In the indirect, the organ (the liver for example) carries from the blood materials charged with carbon, (the bile,) causes the oxygen to predominate in the particles dissolved in the blood of the hepatic veins. The villosities or divisions of the cotyledons of the placenta are formed of tubes terminating in a cul-de-sac in the uterine sinuses. These tubes contain each a ramification of one of the umbilical arteries, which forms a loop by anastomosing with the umbilical vein. This loop represents the capillary net-work of the lungs, or more exactly that of the veins and arteries of the gills of animals which live in the water. These extremities are plunged into the uterine sinuses, from which they are separated by a membrane of great delicacy. The pores of this tunic are large enough to permit the plasma, but not the globules of the blood of the mother, to pass into the placental vessels.

To verify these views, he made the following experiment: After the birth of a child at the Hôtel-Dieu, he tied and cut the cord, and then separated a piece of it, first ligating it at each end. The umbilical arteries presented a deep blue color, the vein a lilac tint. Two punctures were made at the extremities of this loop, one into an artery, the other the vein; the two jets were received on a white surface; that from the artery was of the color of the blood of a vein of an adult; that from the vein was sensibly redder, its color was intermediate to that of arterial and venous adult blood, but approaching venous. The blood by contact with the air became redder. A drop of warm blood from the vein was quickly spread on a warm glass, and, under the microscope, was in every way similar to the blood of an adult. After the separation of the placenta, the umbilical vein presented a lilac reddish tint, while the arteries were much darker, approaching blue.

All subsequent experiments have verified these observations. He concludes from these facts, that the blood of the umbilical vein is more oxygenated than that of the arteries; and that the placenta is an organ for the formation of the blood.

Again, the fœtal bile is the same as the adult, though a little more liquid. Hence, the liver acts the same in the fœtus as the adult. It has for its purpose the decarbonizing of the blood, and giving the child a quantity of that superoxygenated product, glucose. Thus the liver becomes indirectly an organ of sanguification, though it does not play the part of lungs or gills during intrauterine life.

Hence, we may conclude the placenta to be a sort of pediculated gill, an organ of absorption, nutrition, and respiration.—(*Gazette Hebdom.*, February 1, 1861.)

VII.—*Phenomena of Lumbo-Sacral Neuralgia simulating Idiopathic Affections of the Uterus and its Appendages.* By DR. MAROTTE, Paris.

Among the phenomena which are observed in the unimpregnated uterus, the most important is leucorrhœa, a very common affection of cities, and one which is so often connected with disease of the uterus that its neuralgic origin often remains doubtful. It is even notable that in many cases where it is prominent, the neuralgia is not the sole cause—chlorosis, weakness, etc., so favorable to neuralgia, will also produce this discharge. But there are cases where the leucorrhœa is so evidently connected with the pain at its origin, its exacerbation and returns, that no doubt can be entertained.

According to Dr. Marotte, the principal characters of this leucorrhœa are—1st. The sero-mucous or mucous nature of the discharge. The neuralgic leucorrhœa really consists of a transparent, viscous secretion, analogous to a thick solution of gum or starch. 2d. The connection existing between its appearance and its variations in quantity and those of the pains; it seems to increase, or even completely disappear with them. If the patient is subject to a leucorrhœa, the discharge may not cease when the pains disappear, but it always augments with them. The absence of any lesion of the uterus, and the existence of a lumbo-uterine neuralgia, completes the diagnosis.

The special phenomena of the leucorrhœa do not generally demand any other treatment than that for the neuralgia which it accompanies; however, in chronic cases the feebleness causes it to survive the absence of the neuralgia, and then it is necessary to combat it with iron, astringents, or other special agents.

Along with the leucorrhœa there is also an unusual secretion in the genital organs, a gas, of which the nature is not yet determined, and which escapes from the vagina in certain females attacked with lumbo-uterine neuralgia. This is of rare occurrence; it is neither permanent nor abundant like the liquid excretion; it is discharged, from time to time, suddenly, and like an explosion, which relieves the patient, for it is often preceded by uneasiness. Some have this but once; in others, it is repeated. It is necessary to remark here, that Dr. Newcourt has met with females who complained of this expulsion of gas from the vulva, and hence this may occur in the absence of lumbar neuralgia. By touch and a speculum examination, these cases do not generally present any changes in the volume, consistence, or color of the womb; it does not appear to be the seat of any active congestion. Cases are, however, encountered, where the neck is red and swollen, and the lips of the os are introverted. This is seen principally in the acute neuralgias. The congestion spreads even to the vagina and the vulva, if these parts are painful. There is also a chronic form, especially at the time of the inception of the menstrual discharge, and oftener when it is suspended. Dr. Marotte believes that repeated attacks may cause engorgement, hypertrophy, or even a fungous state, which may take the place of the neuralgia.

The congestions are distinguished from the acute stage of metritis by the absence of fever, local heat, the disproportion of the congestive phenomena with the pain, which is sharp, exacerbating, and confined to the nerves of the pelvis and abdomen.

In the chronic state the diagnosis is less easy; however, the absence of heat or any lesions which should be present either in the body or neck in chronic metritis, and finally, the results of the treatment will make it clear.

Another most curious and important peculiarity of this form of neuralgia is metrorrhagia. In some cases the loss of blood is small, and made up of pure blood and sanguineous serosity; these are least numerous, but, at the same time, it is most difficult to connect the hemorrhage with its true origin, because it is generally accompanied by dull pains and without marked twitchings. The loss of blood, sufficiently abundant to constitute true hemorrhages, may form a complication and become a source of weakness by their quantity and duration. Whether abundant or moderate, the metrorrhagia is a special characteristic. The course of these discharges is always irregular, augmenting, diminishing, or ceasing from one day to the next, and often in the same day, without any apparent reason, except their relation with the pains.

The menstrual discharge renews the pains when they have recently ceased, and exaggerates them when present. At the same time the pains often become tenesmoid, which supposes a spasm of fibres of the uterus, and causes a dysmenorrhœa. This is especially seen in young girls and females who lose but little blood, and have never been pregnant, and particularly in chronic cases. In the great majority, this affection exaggerates the menstrua.

In addition to the difficulties of diagnosis, we may have with this neuralgia a hard, frequent, and small pulse, resembling that of peritonitis. In the pregnant state the symptoms are the same, and the hemorrhage, etc. may cause expulsion of the fœtus. This disease may also render the labor very painful and delay the dilatation, when it may cease, or even continue, and produce very severe after-pains.—(*Archives Gen. de Méd.*, Avril et Mai, 1860; *Gaz. Heb.*, September 21, 1860.)

VIII.—*Cauliflower Excrescence of the Os Uteri Removed by the Ecraseur.* By DR. J. BRAXTON HICKS.

The patient, aged forty-seven, of a sallow complexion, and weakened health, had been regular in her functions until the last five months, when the natural discharge was replaced by a watery one, mixed with considerable quantities of blood expelled upon any exertion. She suffered much with pain in the loins. On examination, a growth the size of a small orange was found attached by a pedicle to the posterior lip of the os. It was flattened where it pressed on the rectum, and so soft that the finger could readily penetrate it, causing severe hemorrhage. The operation was performed by bringing the cervix down within the reach of the ecraseur by the double canula and whipcord. The latter was then removed, and the pedicle slowly divided. No hemorrhage or bad symptoms followed. It proved to be a well-marked cauliflower excrescence.—(*Med. Times and Gaz.*, November 17, 1860.)

IX.—*Treatment of Leucorrhœa.* By DR. POCKELS.

In leucorrhœa from a chronic stasis of the uterus, with considerable textural changes or syphilis, Dr. Pockels has found very beneficial results from the use of secale cornutum and catechu; giving of each as much as will lie on the *point of a knife*, three times a day—the catechu being as serviceable as the more expensive tannic acid. If there is anæmia, phosphate of iron is added; and alkalies when acidity of the stomach prevails. An increased secretion of mucus is at first produced, and this may have some blood mixed with it when chronic hydræmia is present.—(*Varges Zeitschrift; Maryland and Virginia Medical Journal*, November, 1860.)

X.—*Treatment of Uterine Inflammation by Injections, External Applications, etc.* By EDW. J. TILT, M.D., London.

Injections are of great value in inflammations of the womb, but require care, in order that their full effect may be obtained. As they are mere lotions to internal organs, the tube of a large syringe, say one or two pints, should be introduced as far as possible without giving pain, in order that the whole lower part of the womb and the entire vagina may be acted on. The patient should be in a reclining position, on a hard sofa, and the liquid injected, and reinjected for at least five minutes; the temperature of the fluid should be warm or tepid in the acute stage, afterward cold. Emollient injections range thus: water, milk and water, linseed tea, solutions of borax, chlorate of potassa, acetate of lead, alum, alum and zinc, zinc alone, decoctions of oak bark, etc. One drachm of the saline compound to a pint of water, and only ten to twenty grains of sulphate of zinc when used alone. Emollients may be used three times a day; cooling twice; antiphlogistics, as alum, only once; astringents two or three times. One drachm of laudanum may be added to each injection when pain is felt. After cure, continue for awhile. Generally, it is best to cease during the menstrual period; but if there is obstinate ulceration, or vaginitis, the medication may be continued. If internal metritis protracts the flow, after it has lasted the usual time, check it by alum and zinc, first tepid and then cold, two or three times a day. Alum injections too long continued, as a preventive of uterine inflammation, have produced irritable subacute inflammation of the os; hence, when astringents are long required, as to enable a relaxed vagina to support the womb, use on alternate days alum and zinc, and acetate of lead. A vagina suppository, to be used at night, may be made with ten grains of acetate of lead and one or two of extract of belladonna. After touching an inflamed cervix with nitrate of silver, it may be *dressed* by pressing up through the speculum one or two tablespoonfuls of starch or rice powder. Externally, in acute inflammations, linseed meal poultices, sprinkled freely with laudanum, may be applied every two hours. Or, half an ounce of an ointment of two drachms of extract of belladonna to an ounce of mercurial ointment may be smeared over the abdomen, and covered with a poultice, both renewed every two hours, while calomel and opium are given internally. Flannels wrung out of hot water, sprinkled with laudanum, and covered with oil-silk, will suit some patients better; or wellheated bags of salt or bran. As inflammation abates, reserve these for the night, or camphorated oil may be rubbed in twice a day and covered with cotton wool. In the chronic stage, foment with hot water, and then apply the cold water compress to the abdomen, covered with oil-silk, renewed when it becomes warm.

*Baths.*—In acute peritonitis, the moving necessary for a warm bath does more harm than good; but this is of great value after the subsidence of the acute symptoms. In acute internal metritis, the hip-bath, at 96° or 98°, for three-quarters of an hour each night before bedtime, quiets pain better than opium. This is useful also in chronic cases and acute affections of the cervix. The whole bath is also invaluable. In all chronic inflammatory conditions of the body and neck of the womb, cold hip-baths are useful, taken immediately on getting out of bed, remaining in two or three minutes, so as to have the benefit of the reaction, without which it is dangerous. In winter the temperature should be 60°. When cold hip or shower baths cannot be borne, sponge alternately with very hot water and water at 60°.

He protests against the use of mercury blindly in obscure cases, and only employs it in syphilitic cases. In hard hypertrophy of the cervix, he prefers iodine and its preparations, both locally and internally.

*Counter-irritants.*—In chronic affections, he produces pustulation of the skin by croton oil or tartar emetic, or cauterizes with the metallic cautery. For sickness attending uterine disease, he applies an issue to the pit of the stomach, curing when all else fails. In catarrhal affections of the cervix, he has found direct blistering useful; rubbing the neck of the womb two or three times with a brush dipped in a concentrated solution of cantharides in sulphuric ether, mixed with the ordinary solution of gutta-percha in chloroform, in the proportion of two parts of the former to one of the latter.

*Dietetics.*—In acute attacks, the horizontal position is imperatively required. But in the chronic form, gentle exercise is requisite, gradually increased.

*Sedatives.*—On this head, he has arrived at these conclusions: an occasional large opiate by the mouth may be useful, but its frequent repetition obscures the case, constipates, and causes opium eating. Sedatives are best administered by the rectum in suppositories or warm milk, thus quelling pain without narcotizing; which would be liable to accelerate the disease. Opiates are advantageously given by the vagina, in suppositories or injections, and hysteralgia is sometimes cured by leaving one or two grains of acetate of morphia in contact with the neck of the womb every third or fourth day. Neuralgic symptoms are often relieved by adding opiates to poultices, ointments, and liniments. Other remedies failing, opiates may be applied to the raw surface of the blistered skin, or injected into the cellular tissue.

*Nitrate of silver, etc.*—It is often necessary to preface this remedy by linseed tea, or other cooling injections. If the solid nitrate increases too much the habitual pains, or causes the ulcerations to bleed for two or three days, it is well to try a solution of forty to sixty grains. This may require a repetition every three or four days. Sometimes he uses a solution in the strength of one ounce of the salt to two or three ounces of distilled water as an application to ulcerated surfaces. Chronic uterine catarrh, or inflammation of the mucous membrane of the cervix, without the slightest abrasion, the membrane of a dusky, livid hue, tender, and secreting pus, may last for years, but generally leads to denudation of the villi, and gives an excoriated appearance. This, with or without excoriation, can be cured by the solution every fourth day, and the solid nitrate occasionally. In obstinate cases, a small portion of the solid stick may be left in the canal.

In vaginitis, the injection is best. The patient should be placed on her back, a small glass speculum introduced as far as possible, and an ordinary glass syringe, full of a solution of forty grains to the ounce, injected. The fluid should be left in contact for five minutes, and then received in a cup. Sometimes as he withdraws the speculum he freely touches the vagina with the solid nitrate diluted by chloride of silver. Especially are these injections applicable in virgins, where a moderate-sized speculum cannot be introduced.

In consequence of the serious accidents following the injection of this article into the womb, he prefers tincture of iodine in solution as an intra-uterine injection. In follicular inflammation of the labia, in eczema and prurigo, or pruritus, external or internal, cotton wool should be soaked in the solution of the nitrate of silver, and carefully rubbed over the diseased surfaces for a few minutes. Even where the disease had existed so long that the pudendal skin felt like parchment, this rubbed in every day, then every other day, then every fourth day, the skin became soft and pliable, and all pain disappeared in a few months.

His experience teaches him that this agent *is not a caustic;* it only condenses tissues, without reducing their bulk. It may cause stricture, but only by this action.

*Tincture of iodine* suits some idiosyncrasies better, and may be used in the strength of one drachm to an ounce of distilled water. It is much less useful than the nitrate of silver.

Ulceration of the cervix may be much shortened by one or two applications of acid nitrate of mercury or potassa cum calce. In fungous and varicose ulceration, these remedies stop the bleeding and promote the cure. In diphtheritic inflammation of these parts, nitrate of silver acts as a poison, but the potassa cum calce effects a cure.—(*Lancet*, Feb. 2 and 23, 1861.)

### XI.—*Chlorosis, especially in Children.* By M. NONAT, of Paris.

Dr. Nonat, from his reflections on the above subject, arrives at the following conclusions:—

Chlorosis is a native, original affection, which precedes functionally a lowering of the blood-making power. It is essentially distinct from anæmia; these two affections differ in their etiology, their alteration of the blood, the progress of the symptoms, and their treatment. Chlorosis constitutes a morbid unity; is always idiopathic. It does not belong exclusively to women; we observe it in men, but less frequently. Far from being the consequence of a suppression or retention of the menses, it is most often the cause of these accidents. We meet with it in all periods of life. It is very frequent in children. It exercises a prejudicial influence on the development of the organism, and plays a great part in the production of diseases, and contributes to lessen their progress and prolong convalescence. Iron is not a specific for this affection, as mercury is for syphilis, and quinine for intermittents. Chlorosis is cured spontaneously with age, in consequence of the regular development of the organism. Nevertheless, it is necessary to give the ferruginous preparations, which constitute, at present, the most efficacious auxiliary medication.—(*Cincinnati Lancet and Observer*, November, 1860.)

